# Dynamic Tuning of MSC-Based Scaffolds for Neurological Protection After Brain or CNS Injury

**DOI:** 10.3390/life16071157

**Published:** 2026-07-13

**Authors:** Mark Slevin, Jerzy Krupinski, Mario Di Napoli, Amelia Tero-Vescan

**Affiliations:** 1 Centre for Advanced Medical and Pharmaceutical Research, George Emil Palade University of Medicine, Pharmacy, Science, and Technology of Târgu Mureş, 540139 Târgu Mureş, Romania; 2 Department of Neurology, Mútua Terrassa University Hospital, 08221 Barcelona, Spain; jkrupinski@mutuaterrassa.es; 3 Neurological Service, SS Annunziata Hospital, 67039 Sulmona, Italy; mario9dinapoli@katamail.com; 4 Biochemistry Department, Faculty of Medicine in English, George Emil Palade University of Medicine, Pharmacy, Science, and Technology of Târgu Mureș, 540142 Târgu Mureș, Romania; amelia.tero-vescan@umfst.ro

**Keywords:** mesenchymal stem cells (MSCs), biomaterial scaffolds, mechanotransduction, YAP/TAZ signaling, neural regeneration

## Abstract

Neurological disorders, including stroke, traumatic brain injury, and spinal cord injury, constitute one of the most important causes of mortality and morbidity worldwide for which current treatment options focus on resolving neuroinflammation rather than on tissue and neuronal regeneration. Mesenchymal stem cells (MSCs) could be a potential therapeutic option due to their immunomodulatory, neuroprotective, and paracrine secretion of extracellular vesicles and trophic factors which modulate microglial activation, preserve blood–brain barrier (BBB) integrity, and neuroplasticity, but with limitations due by poor survival, retention, and phenotypic instability following direct transplantation. The purpose of this narrative review is to present mechanotransduction signaling pathways (integrin–FAK, PI3K/Akt, Rho/ROCK, and YAP/TAZ) through which MSC-based biomaterial scaffolds, especialy hyaluronic acid (HA) hydrogels, make the transition from reparative to regenerative medicine in central nervous system (CNS) injury. Even if most of the evidence from preclinical studies suggests that dynamically tunable MSC-scaffold systems represent promising platforms for neural tissue engineering and regenerative medicine, further translational studies and well-designed clinical investigations are required to establish their therapeutic efficacy and clinical applicability.

## 1. Introduction

Neurological disorders are a major and growing component of noncommunicable diseases globally, accounting for approximately 50% of the top 10 causes of disability-adjusted life years (DALYs) worldwide. According to the most recent Global Burden of Disease (GBD) 2021 study, stroke remains the second leading cause of death worldwide, accounting for approximately 7 million deaths annually, and the third leading cause of death and disability combined, contributing to more than 160 million DALYs [1,2].

Conventional therapies for neuroprotection or post-traumatic injury remain largely symptom-controlling, and whilst they are partially effective at targeting inflammation, and to a lesser degree excitotoxicity, they have not successfully contributed to improvements in repair of the structural microenvironment essential for functional recovery and regeneration of neuronal circuits. Despite substantial advances in stroke rehabilitation, recent large-scale clinical trials have generally failed to demonstrate clinically meaningful improvements in cognitive or motor recovery beyond those achieved with standard rehabilitation. Although participants often showed functional improvement, gains were typically comparable between intervention and control groups, highlighting the ongoing challenges in developing more effective regenerative and rehabilitation strategies., as shown in a recent review by Stinear et al. [3]. Hence, there remains a critical therapeutic gap and a clear need to investigate new regenerative approaches for brain tissue repair.

Mesenchymal stem cells (MSCs) have a proven safety profile whilst exhibiting potential for multipotent differentiation, in addition to phenotypical plasticity, which imparts immunomodulatory properties, and paracrine activity upon them [4]. In rodent models, delivery of MSC-derived exosomes has demonstrated significant improvement in neurogenesis and functional recovery after ischemic stroke, concomitant with reduced infarct volume and improved neurobehavioral changes in rodent models [5]. Similarly, in models of traumatic brain injury (TBI), exosomal administration improved functional recovery whilst increasing neurogenesis, and blocking neuroinflammation, via microglia/macrophage activation [6].

Despite their therapeutic potential, the clinical efficacy of MSCs remains limited by poor cell survival and rapid clearance from the injury site following transplantation into the hostile post-injury microenvironment. For these reasons, biomaterial-based scaffolds have emerged not merely as delivery vehicles, but as essential components of regenerative strategies. The development of biomaterial-based scaffolds has significantly enhanced the therapeutic potential of MSCs by providing them with dynamic and programmable microenvironments. Scaffolds are also able to provide structural support for cell delivery and retention, with experimental studies confirming that MSC+ scaffold transplantation significantly improved cell survival, intercellular communication, and functional recovery in spinal cord injury models [7]. Furthermore, injectable hydrogels loaded with MSC-derived exosomes promoted axonal regeneration, reduced inflammation, and improved neurological function after spinal cord injury, as reviewed in [8]. Dynamic and cell-adaptable hydrogel systems combined with stem cells significantly improved functional repair by modulating the post-injury microenvironment and attenuating neuroinflammation in rats after complete spinal cord resection [9]. The results of these studies suggest that biomaterials recreate key aspects of the extracellular matrix, establishing a permissive niche that supports neuroregeneration and functional recovery after central nervous system (CNS) injuries.

Therefore, in this review, we explore the innovative concept of dynamic regulation and optimization of MSC-based scaffolds in neurological therapy after brain or CNS injuries, highlighting the mechanisms that could change the phenotype, emphasizing strategies in the development of next-generation regenerative systems. Unlike previous reviews that primarily address mesenchymal stem cell therapy or biomaterial scaffolds independently, this review provides an integrated perspective on dynamically tunable MSC-based biomaterial scaffolds as adaptive instructive niches for central nervous system regeneration. In this review, we define dynamic tuning as the MSC-based biomaterial scaffolds with biochemical, mechanical, structural, and temporal properties that can be adjusted to direct MSC behavior according to different stages of CNS repair. Dynamic tuning refers to programmable stiffness and viscoelasticity, controlled presentation or sequential release of bioactive cues, and stimulus-responsive (4D) scaffold adaptations.

## 2. A Brief Overview of MSC Biology in Neurological Contexts

### 2.1. Core Properties of MSCs

MSCs are highly adaptable therapeutic cells well suited for use in the treatment of neurological disorders, considering their capacity to undergo mesodermal differentiation, their immunomodulatory and their paracrine effects. MSCs derived from different tissue sources exhibit distinct biological and therapeutic properties. Bone marrow-derived MSCs (hBMSC) are the most extensively studied and possess strong multilineage differentiation potential, although their proliferation declines with donor age. Adipose-derived MSCs (AD-MSCs) are more easily obtained and display high proliferative and angiogenic capacities, whereas umbilical cord (hUMSCs) and Wharton’s jelly-derived MSCs (WJ-MSCs) exhibit greater proliferative potential, lower immunogenicity, and enhanced paracrine activity. Time-lapse imaging studies have shown that various human MSC populations, e.g., human periodontal ligament-derived stem cells (hPDLSCs), (hBMSCs, and human dental pulp-derived stem cells (hDPSCs), in the appropriate micro-environment, can undergo direct neural differentiation accompanied by active neurite extension and dynamic nuclear remodeling. These processes occur in the absence of mitosis and concomitant with cell dedifferentiation and ultimate acquisition of neuron-like phenotypes [10].

MSCs also have potent immunomodulatory capacity, which could assist in blunting excessive activation of microglia and infiltrating immune cells associated with tissue damage. Recent experimental studies have shown that MSC therapy effectively suppresses neuroinflammation by modulating innate immune signaling. For example, intracerebroventricular transplantation of hUMSCs significantly inhibited microglial pyroptosis in a controlled cortical impact model of TBI. The authors also showed that in vitro, modulation of the tumor necrosis factor-stimulated gene-6 (TSG-6)/NOD-like receptor family pyrin domain containing 3 (NLRP3)/caspase-1/gasdermin D (GSDMD) signaling was responsible for eliciting these effects, in a lipopolysaccharide/ATP-induced BV2 microglial model [11].

Similarly, in amyotrophic lateral sclerosis mouse models, systemic administration of umbilical cord MSC-derived conditioned medium (UCMSC-CM) in SOD1-G93A transgenic mice significantly attenuated microglial activation and astrogliosis. Reduced pro-inflammatory cytokine release and inducible nitric oxide synthase (iNOS) expression were seen in the spinal cord, and the animals showed extended survival times.

In vitro studies using lipopolysaccharide (LPS)-stimulated BV2 microglia further demonstrated suppression of inflammatory signaling and modulation of the microglial secretome, preventing the transition of astrocytes to the neurotoxic A1 phenotype [12].

Together, these findings provide strong evidence for MSCs as active regulators of neuroinflammation rather than passive supportive cells. They consistently demonstrate the capacity to co-ordinate complex immunological and glial responses that define the early reparative phase following neurological injury.

The third major property of MSCs is their paracrine signaling, which is also considered one of the main mechanisms underlying their beneficial neurological effects. MSCs don’t constitute a cell-replacement therapy, instead, they release a complex secretome composed of cytokines, growth factors, lipids, metabolites, and extracellular vesicles, including exosomes, which influence neuronal survival, glial activation, synaptic remodeling, and tissue repair [13,14]. In ischemic stroke, exosomes derived from hUMSCs carrying miR-146a-5p were shown to be internalized by neural cells at the site of injury and to attenuate microglia neuroinflammation both in vivo and in oxygen and glucose deprivation-induced microglial models in vitro, through suppression of the interleukin-1 receptor-associated kinase 1 (IRAK1)/TNF receptor-associated factor 6 (TRAF6) signaling pathway, resulting in reduced infarct volume, decreased microglial activation, and improved neurological and behavioral recovery in mice [15]. Despite these promising findings, the clinical translation of EV-based therapies remains challenging due to the lack of standardized isolation and purification protocols, heterogeneity in EV composition, limited scalability for large-scale production, and uncertainties regarding optimal dosing, biodistribution, and long-term safety. Furthermore, variations in EV cargo related to MSC tissue source and culture conditions may affect therapeutic reproducibility, highlighting the need for standardized manufacturing procedures and well-designed clinical studies before EV-based therapies can be routinely implemented in neurological regeneration.

In vitro, exosomes derived from hUMSCs promoted functional recovery in 6-hydroxydopamine (6-OHDA; Parkinson-like model)-SH-SY5Y-injured cells following their internalization and restoration of normalised autophagy, with a clear reduction in cell apoptosis. In vivo, systemically administered MSC-derived exosomes were able to cross the blood–brain barrier (BBB), in rats, with IHC studies demonstrating localization within the substantia nigra, and co-localization with dopaminergic neurons. Repeated intravenous administration resulted in reduced neuronal loss and improved motor function as determined by a battery of behavioural studies [16].

The phenotypical multipotency and plasticity of MSCs, together with their highly bioactive secretome, therefore make them ideal as a potential therapeutic in scaffold-based neurological treatments. In this case, environmental instruction can bias MSC behavior toward either reparative or regenerative programs, creating novel and dynamic (and tunable) micro-environmental niches.

### 2.2. Reparative Phenotype of MSCs

The reparative phenotype of MSCs is activated during the acute, early phase of injury and is aimed at attenuating the inflammatory milieu characterized by excessive immune activation, oxidative stress, and cell death, primarily involving microglia and infiltrating macrophages.

MSCs can reduce cytokine levels and also actively interfere with damage-associated signaling cascades, particularly inflammasome activation and pyroptotic cell death. For example, intracerebroventricular administration of hUMSCs 4 h post-injury significantly suppressed the NLRP3/caspase-1/GSDMD signaling pathway whilst improving neurological outcome in a controlled cortical impact model in BALB/c mice. These effects were mediated by the anti-inflammatory factor TSG-6, since shRNA-mediated knockdown of TSG-6 in hUMSCs markedly reduced their therapeutic efficacy. Recombinant TSG-6 administration reversed the anti-pyroptotic effects. Complementary in vitro experiments using lipopolysaccharide/ATP-stimulated BV2 microglial cells further confirmed that MSC-derived TSG-6 directly inhibited inflammasome activation and microglial pyroptosis [11].

In parallel, MSCs present cytoprotective effects on neural and glial populations by modulation of intracellular survival pathways, including anti-apoptotic signaling and mitochondrial stabilization, which together limit neuronal loss in the acute phase of injury. Importantly, these effects are not restricted to neurons but extend to astrocytes and endothelial cells, thereby contributing to broader tissue preservation. In a rat model of spinal cord ischemia–reperfusion injury, bone marrow–derived MSC exosomes administered intrathecally significantly improved hind-limb locomotor function. blood–brain barrier (BBB) scoring, reduced histopathological damage, and attenuated neuronal degeneration in lumbar spinal cord segments were all noted. These effects were further enhanced by the incorporation of miR-455-5p within the exosomes, which reduced cellular apoptosis and neuronal death, supporting a direct cytoprotective role of MSC-derived extracellular vesicles in vivo [17].

Early BBB disruption is a major driver of edema, and it leads to enhanced immune cell recruitment, and progressive tissue degeneration. In this regard, MSCs have been shown to maintain structural and functional integrity of the CNS microenvironment by stabilizing the neurovascular unit, including the BBB. MSCs significantly reduced BBB permeability in a controlled cortical impact model of traumatic brain injury. Their systemic administration induced upregulation of tissue inhibitor of metalloproteinase-3 (TIMP3) and subsequent inhibition of matrix metalloproteinase (MMP) activity. In parallel, in vitro co-culture of MSCs with primary endothelial cells reduced tumor necrosis factor alpha (TNF-α)–induced endothelial permeability, further supporting a direct role of MSCs in preserving endothelial barrier integrity [18].

The available evidence therefore indicates that the primary role of MSCs is to limit the progression of tissue injury rather than directly replace damaged cells. By modulating inflammation and preserving the surrounding tissue, MSCs can seemingly create a neo-microenvironment that supports endogenous repair and functional recovery.

### 2.3. The Regenerative Phenotype of MSCs and the Benefits of Plasticity

The regenerative phenotype of MSCs emerges in later phases of injury and is characterized by active promotion of tissue reconstruction, neural plasticity, and functional recovery, showing a unique microenvironmental reprogramming rather than a basic damage control strategy [19].

A central of regenerative mechanism of MSC is providing trophic support that enhances endogenous repair mechanisms, such as via stimulation of resident neural progenitors, support of synaptic plasticity, and facilitation of structural remodeling. In a nonhuman primate (Rhesus monkey) model of cortical injury, bone marrow MSC-derived extracellular vesicles enhanced neural circuit remodeling by reducing perilesional neuronal hyperexcitability, restoring excitatory/inhibitory balance, and increasing apical dendritic branching complexity and spine density in ventral premotor cortical neurons. These changes were correlated with improved hand function and recovery, indicating fine motor control improvement [20].

More recently, MSC-derived extracellular vesicles have been shown to promote neurogenesis and synaptic remodeling in experimental models of brain injury. For example, in a murine neonatal BALB/c model of cerebral palsy (hypoxia-ischemia +LPS), neuronal damage was reduced following administration of hUMSCs exosomes. Concomitantly, the treatment restored normal synaptic ultrastructure in the hippocampus. These effects were accompanied by a marked increase in the expression of the synaptic proteins, synaptophysin and postsynaptic density protein 95 (PSD-95), which intigated increased pre- and postsynaptic integrity. Notably, combined treatment with exosomes and nerve growth factor further enhanced these effects, indicating a synergistic trophic mechanism [21].

MSC can also modulate neuron–glia interactions, where they support functional axonal regeneration and circuit reorganization. This is achieved mechanistically through effective promotion of the transition from the existing proinflammatory environment toward a repair-oriented niche. Suppression of the neurotoxic astrocyte phenotypes together with enhancement of supportive glial functions effectively facilitates synaptogenesis and axonal growth. For example, i.v. administration of MSC exosomes (isolated from rhesus monkey bone marrow) significantly enhanced remyelination in a mouse model of cuprizone-induced and experimental autoimmune encephalomyelitis. The treatment was able to promote oligodendrocyte progenitor cell differentiation, whilst increasing the proportion of mature oligodendrocyte populations (BrdU^+^/APC^+^ and APC^+^), and at the same time elevate myelin basic protein expression. In addition, the presence of MSCs modulated neuron–glia interactions, through a toll-like receptor 2 (TLR2)/IRAK1/nuclear factor kappa-light-chain-enhancer of activated B cells (NF-κB)-dependent mechanism which resulted in suppression of pro-inflammatory M1 microglia, whilst enhancing M2 polarization [22].

The data therefore suggests that the regenerative phenotype may be a dynamic state of MSC that switches on in response to a permissive microenvironment. Although the distinction between reparative and regenerative MSC phenotypes is not yet universally accepted, it is useful for describing the functional plasticity of MSCs during tissue repair. In particular, MSC-derived EVs, including exosomes, are able to promote neuroplasticity and synaptic remodeling in conjunction with stimulation of endogenous repair mechanisms. Current preclinical evidence suggests that these processes may contribute to improved functional and cognitive recovery following neurological injury; however, further translational and clinical studies are required to validate their therapeutic efficacy.

## 3. Incorporation of Biomaterial Scaffolds as ‘Instructive Niches’ in Neurological Repair

Consistent evidence has demonstrated that biomaterial scaffolds will form a critical component of next-generation regenerative neuro-therapy. In addition to enabling passive carriege for MSC transplantation, they enable construction of dynamic and bioinstructive microenvironments with the capacity to regulate cell behavior, inflammation, and tissue remodeling following CNS injury. Through modulation of multiple (e.g., biochemical, mechanical, electrical, and topographical) cues, they positively influence MSC survival, phenotype switching, and therefore overall paracrine activity, whilst directing neural lineage commitment. These features, make them ideally suited to drive the transition from early neuroprotection toward long-term regeneration. Evidence now suggests that scaffold architecture and composition may be equally as important as the transplanted cells themselves, particularly in the hostile post-injury microenvironment, with dysregulated stress and inflammatory pathways together with a complete extracellular matrix disruption. Hence, in this section, the major scaffold systems will be explored for neurological applications and consideration given to how scaffold–cell interactions may be strategically tuned to optimize neurorepair and functional recovery after CNS injury.

### 3.1. Types of Scaffolds Suited to MSC Incorporation

Biomaterial scaffolds are now considered critical components in neural tissue engineering, where they can be tuned to regulate stem cell fate, modulate inflammation, and guide tissue regeneration. In the CNS, the natural capacity for intrinsic cellular and tissue regeneration is limited. However, specific scaffold composites, together with their architecture and physicochemical properties, can be designed to modulate MSC responses towards neurogenesis, angiogenesis, and axonal regeneration.

Hydrogels are among the most widely used scaffolds in CNS applications due to their high-water content and similarity to native extracellular matrix (ECM). These have been shown in several model systems to effectively mimic the brain ECM. For example, vascular endothelial growth factor (VEGF)-loaded chitosan–hyaluronic acid (HA) hydrogel containing rat adipose-derived stem cells (ADSCs), when stereotactically injected into the peri-ischemic region 24 h after transient middle cerebral artery occlusion (tMCAO) in rats, significantly reduced infarct volume and neuronal apoptosis. The ADSCs showed enhanced ADSC viability and paracrine activity, partially due to increased expression of fibroblast growth factor 2 (FGF-2), transforming growth factor beta (TGF-β), brain-derived neurotrophic factor (BDNF), and insulin-like growth factor 1 (IGF-1).

Concomitantly, ADSCs protected primary cortical neurons from oxygen-glucose deprivation/reoxygenation injury by reducing apoptosis through enhanced Bcl2 and reduced cleaved caspase-3 expression, restoring mitochondrial membrane potential, and preserving axonal morphology [23]. We will discuss the use of HA in more detail in a later section.

Under combined biochemical induction and structural guidance, nano-structured GelMA (gelatin methacryloyl) hydrogels incorporating Wharton’s Jelly-derived mesenchymal stem cells (WJ-MSCs) exhibited enhanced neuroectodermal commitment. Cell signalling demonstrated concomitant over-expression of SRY-box transcription factor 2 (Sox2) and paired box gene 6 (Pax6), together with enhanced calcium signaling and voltage-responsive electrical activity. This showed that scaffold topography could actively drive MSC neural differentiation toward specific functional phenotypes [24].

Similarly, graft survival of glial cell line-derived neurotrophic factor (GDNF)-overexpressing bone marrow-derived MSCs isolated from GFP-transgenic rats in normal rat striatal parenchyma was significantly enhanced after ‘gelling’ with type I collagen hydrogel. Cell viability improved alongside enhanced neurotrophic factor secretion in vitro, whilst in vivo, a reduction in both microglial activation and astrocyte recruitment at the graft site, was seen in treated animals. These findings indicated that reduction in local inflammation significantly improved MSC integration within the brain [25].

In contrast, electrospun nanofibers are able to mimic the aligned architecture of neural tissue, providing topographical cues that can guide axonal growth. For example, hUMSCs cultured on electrospun polypyrrole/polylactide (PPy/PLA) composite nanofiber films exhibited significantly enhanced neurogenic differentiation through a mechanism involving increased expression of the neuronal markers nestin and neurofilament, compared to cells grown on random or non-stimulated substrates [26]. Hence, appropriate electrical stimulation combined with the integration of properly aligned topography synergistically directed MSC fate toward electro-responsive neuronal phenotypes. Most recently, graphene-based conductive nanointerfaces have also been used as bioactive instructive platforms, able to promote both structural and functional neuronal commitment of MSCs.

This was shown for example in hBMSCs cultured on gelatin-coated substrates functionalized with graphene nanoplatelets (GNPs). Here, the cells presented significantly increased expression of the key neuronal markers microtubule-associated protein 2 (MAP2), Nestin, and βIII-tubulin (Tuj1), accompanied by morphological changes indicative of neuron-like phenotypes and increased intracellular calcium signaling [27]. This and other recently published studies therefore support the use of such conductive nanofiber scaffolds as intuitive platforms suitable for neural tissue engineering.

For decades we have seen that direct transplantation of MSCs (without matrix incorporation) is limited by poor survival and, probably for this reason, has rarely shown any positive remodelling effects in human disease trials or studies, with even only a modest impact in pre-clinical rodent models. However, data derived from similar animal models using these novel (described above) enhanced ‘graft-like’ combinations has to date been far more promising.

For example, implantation of 3D-printed collagen/silk fibroin scaffolds loaded with MSC-derived secretome at the site of a T10 spinal cord transection (Sprague–Dawley rat model) significantly improved functional recovery and promoted nerve fiber regeneration and remyelination, concomitant with a notable improvement in synaptic connectivity. The animals showed physical improvements confirmed with behavioral and electrophysiological analysis [28].

Similarly, Zheng et al. created a polydopamineimidazole-modified gelatin methacrylate gel containing stromal-cell-derived-factor-1 and human amniotic-derived MSCs, which successfully supported regeneration of endogenous nerve cells following injection into a cryogenically-induced parietal injury in a rodent model [29]. In a separate study, programmable shape (3D printed) memory sericin scaffolds were successfully implanted in mice following transient MCAO, filling the exact shape of the infarct as shown by micro-CT imaging and delivering bone marrow-derived MSCs that self-differentiated into neural (MAP2-positive) precursors [30]. The most relevant studies on biomaterial scaffolds published in recent years are summarized in Table 1.

**Table 1:** Key studies presenting biomaterial scaffolds as instructive niches regulating MSC behavior in CNS regeneration.

Scaffold Type	Biomaterial	Cell Type/Model	Experimental Context	Biological Effects	Main Outcomes	Ref.
Hydrogel (growth factor-loaded)	Chitosan–hyaluronic acid + VEGF	Rat ADSCs/tMCAO model	In vitro + in vivo	↑ Paracrine factors (BDNF, IGF-1); ↓ apoptosis; mitochondrial stabilization	↓ Infarct volume; improved motor recovery	[23]
Hydrogel (topography-driven)	Micro/nano-structured GelMA	WJ-MSC spheroids	In vitro	↑ Sox2, Pax6; Ca^2+^ signaling; neuronal activity	Functional neuron-like phenotype	[24]
Hydrogel (cell delivery)	Type I collagen	GDNF-MSCs; rat brain	In vitro + in vivo	Maintained viability ↓ microglia and astrocytes activation ↓ astrocyte recruitment at the graft site	Improved graft integration	[25]
Electrospun nanofibers	Ppy/PLA	hUC-MSCs	In vitro	↑ Nestin, neurofilament	Enhanced neurogenic differentiation	[26]
3D-printed scaffold	Collagen/silk fibroin + secretome	Rat SCI model	In vivo	↑ axonal regeneration; ↑ remyelination	Improved locomotor recovery	[28]
Conductive nanointerface	Graphene nanoplatelets	hBMSCs	In vitro	↑ MAP2, Tuj1; ↑ Ca^2+^ signaling	Enhanced neuronal maturation	[27]

Abbreviation: ADSCs—adipose-derived stem cells; SCI—spinal cord injury, VEGF—vascular endothelial growth factor, BDNF—brain-derived neurotrophic factor, IGF-1—insulin-like growth factor 1, Sox2—SRY-box transcription factor 2, Pax6—paired box gene 6; GDNF-MSCs—glial cell line-derived neurotrophic factor-modified MSCs, Ppy/PLA—polypyrrole/polylactic acid, hUC-MSCs—human umbilical cord-derived MSCs, MAP2—microtubule-associated protein 2, Tuj1—βIII-tubulin, hBMSCs—human bone marrow-derived MSCs, ↑—up arrow means increase, ↓—down arrow means decrease.

### 3.2. Mechanistic Analysis of Scaffold–Cell Interactions

As we have seen already above, interactions between biomaterial scaffolds and MSCs is a requirement for regeneration in neural tissue. Scaffolds provide structural support, and instructive microenvironments that are necessary to promote optimal adhesion, mechanical, topographical, and biochemical signals. The following elements are/would be considered critical for their optimal translation and use.

#### 3.2.1. MSC Adhesion

MSC adhesion is based on the interaction of integrins with the ECM, which triggers a variety of cellular signaling cascades culminating in promotion of their survival and differentiation. For example, collagen-based hydrogels with a structure similar to the ECM have demonstrated enhanced retention, survival, and therapeutic efficacy of MSCs. In this way, they provide a permissive 3D microenvironment that should facilitate cell–matrix interactions and nutrient diffusion. Confirming this, a recent study using human bone marrow-derived MSCs embedded in dermis-derived atelocollagen hydrogels showed amplified immunomodulatory gene expression and increased matrix contraction under inflammatory conditions. These effects were determined by collagen concentration and cell density, with increasing elastic modulus and reduced contraction correlating with increased collagen, suggesting that optimizing matrix parameters may enhance the therapeutic response of MSCs [31].

Integrin-dependent adhesion is crucial for MSC survival and differentiation. For example, cytoskeletal organization, and enhanced lineage commitment of co-cultured rat embryonic neural stem cells with MSC femoral-derived spheroids in a collagen matrix was dependent upon activation of focal adhesion kinase (FAK) and downstream associated mechanotransduction pathways (phosphoinositide 3-kinase (PI3K); protein kinase B (Akt); Ras homologous GTPase (Rho)/Rho-associated coiled-coil containing protein kinase (ROCK) [32].

#### 3.2.2. Matrix Stiffness

Matrix stiffness and biochemical modifications particularly collagen oxidation or glycation significantly influence MSC mechanotransduction and differentiation. As we have just described, mechanotransduction relies on the integrin–FAK axis and this activates downstream Yes-associated protein (YAP)/transcriptional co-activator with PDZ-binding motif (TAZ) signaling, as determinants of ECM compliance and topography. Mechanically compliant or disrupted collagen substrates are able to alter cytoskeletal tension, modulating focal adhesion maturation, and confering nuclear localization of YAP/TAZ. This acts as a type of master switch that defines MSC differentiation and imunomodulatory function.

Recent experimental studies have shown that mechanical forces and matrix composition jointly dictated the formation and maturation of 3D-matrix adhesions (3DMAs), integrin αV/α5 binding, and FAK–YAP signaling in bone marrow MSCs cultured within collagen microtissues under dynamic unidirectional compression loading [33]. Oxidation and glycation of collagen also modified its nanotopography and fibrillar organization, impairing MSC focal adhesion development and altering YAP/TAZ activation patterns during early adhesion, as demonstrated by Komsa-Penkova et al. in adipose tissue-derived MSCs [34]. These results demonstrate that perturbations in collagen structure, whether chemical (glycation, oxidation) or mechanical (stiffness changes, can disrupt mechanosensitive feedback loops essential for lineage specification and cell survival.

Furthermore, culture of adipose-derived MSCs within N-cadherin–mimetic 3D hydrogels imparted the capability for them to differentiate into pro-regenerative and immunomodulatory states whilst maintaining stemness as demonstrated through continued expression of CD90/CD105 and upregulation of β-catenin, MMP-2, and vascular endothelial growth factor receptor 2 (VEGFR2). The study strongly supported the view that this scaffold/micro-environment could provide bioinstructive physical and biochemical cues that complemented ECM mechanotransduction [35].

Together, these studies demonstrated that both matrix stiffness and microstructural modifications govern MSC adhesion signaling through integrin and cadherin crosstalk, ultimately shaping their reparative, neuroprotective, and immunoregulatory responses in engineered 3D environments.

#### 3.2.3. Hyaluronic Acid (HA)—A Hypothetical but Potentially Effective Neural Scaffold Design?

HA may be of particular interest in neural tissue engineering because it represents one of the principal ECM components of the brain. Here it stabilises the ECM of cerebral tissue contributing significantly to hydration and viscoelasticity, However, in addition, it also supports (and possibly drives) neuronal migration, synaptic plasticity and mechanotransduction, whilst its strong natural anti-inflammatory capacity is able to dampen excessive neuroinflammatory responses [36].

Unlike many peripheral tissues, the CNS ECM is relatively poor in fibrillar collagen and instead highly enriched in glycosaminoglycans such as HA. HA is found in high concentrations particularly within perineuronal nets and neurogenic niches, where it interacts with proteoglycans, tenascins, laminin, and cell-surface receptors including cluster of differentiation 44 (CD44) and receptor for hyaluronan-mediated motility (RHAMM), to regulate neural cell behavior. As such, it may be a critical driver normalizing the ECM after tissue damage following stroke or traumatic brain injury (as eloquently reviewed by Shahi et al.) [37].

Importantly, the biological effects of HA are highly dependent upon molecular weight. High molecular weight (HMW) and ultra-high molecular weight (UHMW; as seen in the naked mole rat) HA generally exerts anti-inflammatory, neuroprotective, anti-fibrotic, and homeostatic functions [38]. In contrast, fragmented low molecular weight (LMW) HA can act as a damage-associated molecular pattern (DAMP) capable of activating TLR2/4-mediated inflammatory signaling, although one recent study by Jia et al., actually showed that collagen tissue scaffolds containing LMW HA improved calvarial bone repair in male Sprague-Dawley rats through stimulation of pro-angiogenic activity, indicating that in certain ‘inflammation-protected’ micro-environments, the fragments could also have reparative properties [39]. Therefore, HMW/UHMW HA may provide a uniquely favorable scaffold environment, limiting astroglial/glial hyperactivity and scar formation, whilst preserving BBB integrity.

At the same time, HA-rich matrices support MSC survival, migration, and paracrine signaling, whilst facilitating extracellular vesicle production, controlling diffusion of neurotrophic factors and extracellular vesicles throughout the injured tissue [40]. The issue too date is that most of the currently published work refers to their incorporation at sites of joint injury or cartilage damage, where literally hundreds of publications over the last five years or so have described the use of HA-MSC scaffolds for protecting and restoring joint cartilage. For example, Zhou et al. recently showed that human umbilical cord-derived MSCs implanted within a methacrylate-HA hydrogel, produced a significant improvement in repair in an osteochondral murine defect model, whilst concomitantly upregulating SRY-box transcription factor 9 (Sox9), hypoxia-Inducible Factor 1 alpha (HIF-1α), and collagen type II (COL2) in a consolidating hypoxic microenvironment [41].

So, HA-based hydrogels can dynamically regulate MSC behavior through both biochemical and biomechanical signaling. HA influences integrin-independent mechanotransduction via CD44-mediated cytoskeletal remodeling and can modulate YAP/TAZ activity through alterations in matrix stiffness and viscoelasticity. In addition, HA hydrogels, covalently cross-linked with methacrylated scaffolds, exhibit very high injectability and a shear-thinning behavior. This extreme tunable degradation kinetics make them particularly suitable for minimally invasive intracerebral or intrathecal delivery [42].

Incorporation of ultra-high molecular weight HA, (which has extreme stability to breakdown) into multifunctional scaffolds may be especially advantageous for recreating the highly hydrated and mechanically compliant neural ECM, thereby establishing a permissive microenvironment for neurogenesis, axonal remodeling, remyelination, and long-term circuit reconstruction. In this respect, methacrylated gelatin cross-linked to UHMW showed superior tensile strength, cellular activation, and osteogenic differentiation of rabbit bone-marrow-derived MSCs in vitro, together with runt-related transcription factor 2 (RUNX-2) and bone morphogenetic protein 2 (BMP-2) overexpression in a FAK-integrin-dependent mechanism [43].

Hence, the published data indicate that HA may function as a temporally dynamic instructive cue within “smart” regenerative biomaterials. The current evidence suggests that early after injury, HA-rich matrices are capable of buffering inflammatory signaling and oxidative stress, whereas in later phases they support synaptic stabilization, neuronal plasticity, and endogenous progenitor migration.

The incorporation of HMW or UHMW-HA (as seen in the naked mole rat which *incredibly* lives for over 30 years!) [44,45] within multifunctional scaffolds may be particularly advantageous following CNS injury. As described earlier, these HA polymers closely mimic the hydrated native brain ECM, providing authentic viscoelastic biomechanical support. In addition, they provide a protective permissive microenvironment that minimises glial scar formation associated with aging or infarction, whilst preserving tissue hydration, which assists neurogenesis, and associated neural circuit remodeling after brain injury [46].

**Figure 1:**
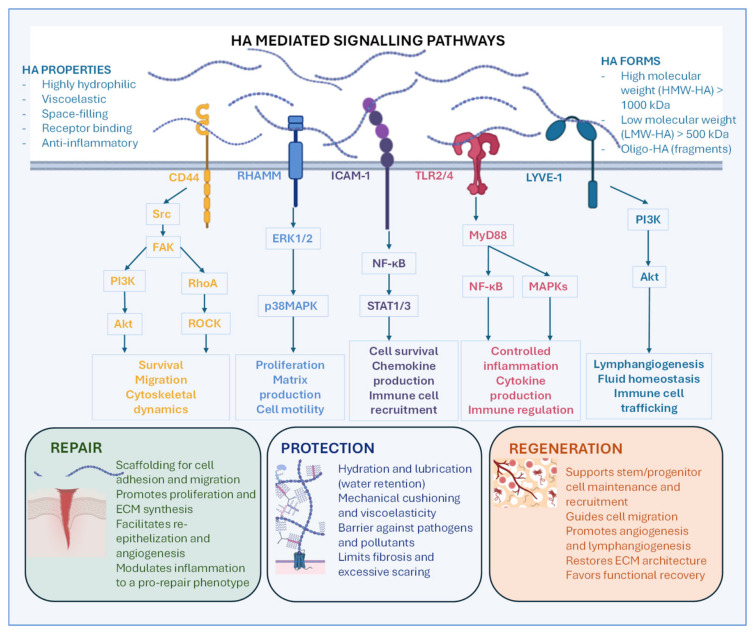
HA-based “smart” neural scaffolds as neuroprotective and neuroregenerative after CNS injury (stroke, traumatic brain injury or spinal cord injury). HA is a major component of the neuronal ECM that interacts with CD44, RHAMM, integrins, proteoglycans, laminin and tenascins. HMW/UHMW-HA exerts predominantly anti-inflammatory, neuroprotective, antifibrotic and homeostatic effects, including suppression of microglial hyperactivation, attenuation of reactive gliosis and glial scar formation, preservation of BBB integrity and support of MSC survival, migration and paracrine signalling. Abbreviations: HA—hyaluronic acid, CNS—central nervous system, HMW—high molecular weight and UHMW—ultra-high molecular weight HA, ECM—extracellular matrix, CD44—cluster of differentiation 44, RHAMM—receptor for hyaluronan-mediated motility, BBB—blood–brain barrier, FAK—focal adhesion kinase, ICAM-1—intercellular adhesion molecule 1, TLR2/4—toll-like receptor 2/4, LYVE-1—lymphatic vessel endothelial hyaluronan Receptor 1, Src—proto-oncogene tyrosine-protein kinase, PI3K—phosphoinositide 3-kinase, Akt—protein kinase B, RhoA—Ras homolog family member A, ROCK—Rho-associated coiled-coil containing protein kinase, ERK1/2—extracellular signal-regulated kinase 1/2, p38MAPK—p38 mitogen-activated protein kinase, NF-κB—nuclear factor kappa-light-chain-enhancer of activated B cells, STAT1/3—signal transducer and activator of transcription 1/3, MyD88—myeloid differentiation primary response 88, MAPKs—mitogen-activated protein kinases. Created in BioRender. Tero-Vescan, A. (2026) https://BioRender.com/w1661di.

Furthermore, HA serves as an ideal platform for combination therapies, including incorporation of MSC-derived exosomes, growth factors (BDNF, NGF, VEGF), conductive nanomaterials, or lyophilised microfragmented adipose tissue (MFAT), thereby enabling sequential and feedback-responsive delivery systems. Such HA-enriched “smart” scaffolds could therefore act as dynamically tunable regenerative matrices capable of shifting the post-injury environment from inflammation containment toward long-term structural and functional neural repair. In this regard, Li et al. created an MnO_2_—nanoparticle dotted HA hydrogel which supported tissue bridging of injected MSCs and demonstrated spinal tissue integration with neural differentiation associated with motor functional recovery in a model of rat spinal cord transection [47]. Directly relating to brain injury and to date (May 2026), we have only identified one publication that utilised a HA scaffold in the context of stroke. In this study, the authors constructed a scaffold of HA and chitosan, loaded with VEGF and adipose-derived rat MSC, delivering it by direct stereotactic injection to the infarcted region of the brain following transient MCAO. Compared with sham controls, neuronal survival, mitochondrial membrane potential, and axon morphology were preserved concomitant with improved motor function and lower morbidity, although it should be noted that the authors did not directly compare the effectiveness of this scaffold with other derivatives [23]. A summary of the anticipated effects and mechanisms of action of Hais is provided in the Figure 1 below.

Future studies should focus on the development of dynamically tunable HMW/UHMW-HA-based multifunctional neural scaffolds capable of temporally reorganizing anti-inflammatory, neuroprotective, angiogenic, and regenerative signaling. This can be achieved through combining their mechanical properties, extracellular vesicle delivery, and reconstruction of the endogenous neurogenic niche. The development of such next-generation scaffolds could limit secondary CNS injury and actively promote long-term structural and functional brain repair through coordinated actions we have described above.

#### 3.2.4. Topographical Cues Provide Critical 3D Alignment Within the Brain Microenvironment

Topographical cues are an im portant biomechanical factor which guide MSC orientation, cytoskeletal alignment, and lineage specification. Scaffold anisotropy, co-ordinated by mechano-signalling mimicks the hierarchical organization of the ECM and is critical in directing neurite outgrowth and promoting neuronal differentiation.

Micro- and nano-patterned hydrogels can inherently prime MSCs toward a neurogenic fate even in the absence of exogenous growth factors. For instance, as detailed earlier in this review, Maassen et al., showed that GelMA hydrogels with microgrooved topographies seeded with Wharton’s jelly-derived MSC-spheroids showed enhanced ectodermal trans-differentiation [24]. Similarly, ECM-mimicking aligned electrospun collagen or polymeric nanofiber scaffolds have been shown to promote cytoskeletal organization and neurite-oriented growth in MSCs, concomitant with increased expression of the neuronal maturation markers βIII-tubulin, nestin, and MAP2, suggesting accelerated neural lineage commitment and structural maturation [48].

Moreover, complementary experimental data from human bone marrow-derived MSCs cultured on compressed collagen type I matrices demonstrate that substrate density and microstructure critically influence MSC adhesion, proliferation, and cytoskeletal organization. In these models, compressed collagen scaffolds derived from rat tail tendons provided aligned fibrillar topographies that guided MSC elongation and modulated focal adhesion formation, confirming the importance of collagen anisotropy in regulating cellular mechanobiology [49].

Evidence provided in the above section supports strongly the importance of topographic cues as key regulators of MSC co-ordinated activities. Taking into account the changes in biochemical parameters that have been observed, the production and utilization of next-generation neuronal regenerative cells should be achievable.

#### 3.2.5. Biochemical Functionalization and Controlled Signaling of Neurological Scaffolds

Biochemical functionalization enables scaffolds to deliver growth factors, cytokines, or secretome components that recreate a pro-regenerative microenvironment and support neural repair. For example, as described earlier, incorporation of only the MSC secretome into 3D-printed collagen/silk fibroin scaffolds (rather than the cells) in a rat T10 spinal cord transection injury model enabled significant axonal regeneration, and re-myelination. Thes effects were obtained purely from sustained local release from the scaffold of neurotrophic and immunomodulatory factors [28].

Complementary evidence showed that electroconductive amino-functionalised collagen–graphene cryogels, were capable of promoting M2 (anti-inflammatory CD206/CD163-positive) macrophage polarization and enhanced expression of βIII-tubulin and MAP2 in bone marrow-derived MSCs. Enhanced neuronal differentiation under electrical stimulation was considered to be due to greater scaffold structural stability [50].

Electrical responsiveness of bone marrow-derived MSC was also enhanced in graphene nanoplatelets supported within collagen/gelatin gels [27]. Similarly, laminin-conjugated aligned nanofiber yarns (incorporating N-hydroxysuccinimide ester groups) were shown to support co-ordinated fasciculated neurite bundle formation, and reduced aberrant branching in PC12 dorsal root ganglion cultures. In this way, the gel provided dual topographical and biochemical guidance that enabled long-range directional neurite outgrowth. This experimental data clearly illustrated the beneficial synergy between spatial architecture and biochemical signaling [51].

These studies provide clear evidence that integrating biochemical functionalization with conductive and aligned architecture is able to create bioinstructive scaffolds that can respond to (and coordinate) trophic, electrical, and mechanical cues. Incorporation of these clinically or therapeutically could help to optimize MSC-driven neuroregeneration following brain trauma. However, a limitation to date of these studies to date is the lack of confirmation in vivo pre-clinical models, and this needs to be addressed.

## 4. Dynamic Reciprocity in MSC–Scaffold Systems

The principle of dynamic reciprocity recognizes that cells and the ECM continuously influence one another. The ECM provides mechanical support but in addition, it regulates key cellular functions, including adhesion, migration, proliferation, differentiation, and survival. In response to these signals, cells actively remodel their surrounding matrix, allowing the tissue microenvironment to adapt during development, repair, and disease [52]. In the context of MSC neural repair, this reciprocal relationship forms the mechanistic foundation for how engineered scaffolds can provide better regenerative outcomes in the injured CNS [53].

### 4.1. Integrin-Mediated Signaling

**Figure 2:**
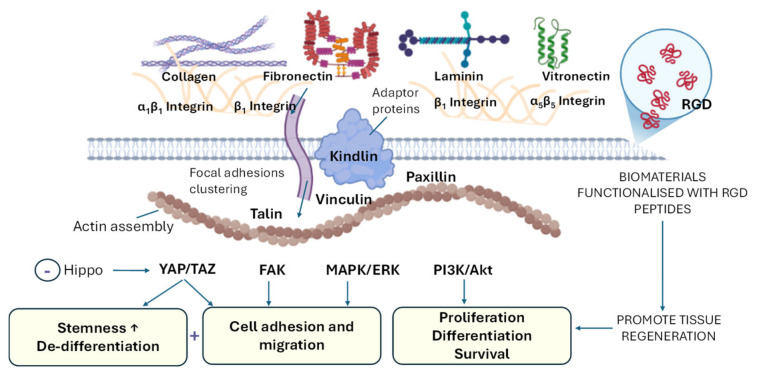
Integrin-mediated mechanotransduction and downstream signaling pathways involved in tissue regeneration and stem cell plasticity. ECM proteins, including collagen, fibronectin, laminin, and vitronectin, interact with specific integrins (α1β1, β1, and α5β5 integrins), promoting integrin activation and focal adhesion clustering. Intracellular adaptor proteins such as talin, kindlin, vinculin, and paxillin connect integrins to the actin cytoskeleton and regulate focal adhesion assembly. These interactions activate key signaling pathways, including FAK, MAPK/ERK, PI3K/Akt, and YAP/TAZ signaling through Hippo pathway modulation. Downstream effects include enhanced cell adhesion, migration, proliferation, differentiation, survival, stemness, and cellular dedifferentiation. In parallel, biomaterials functionalized with RGD peptides further enhance integrin signaling and regenerative responses by improving cell–material interactions and cytoskeletal organization. Abbreviations: ECM—extracellular matrix, FAK—focal adhesion kinase, MAPK—mitogen-activated protein kinases, ERK—extracellular signal-regulated kinase, PI3K—phosphoinositide 3-kinase, Akt—protein kinase B, YAP—Yes-associated protein, TAZ—transcriptional co-activator with PDZ-binding motif, RGD—the one-letter amino acid sequence abbreviation for Arg–Gly–Asp. Created in BioRender. Tero-Vescan, A. (2026) https://BioRender.com/iih3868.

Integrins are the primary mechanochemical transducers linking the ECM to intracellular signaling cascades. They are heterodimeric transmembrane receptors that recognize specific ECM ligands (collagen, fibronectin etc.), and transmit information bidirectionally across the plasma membrane. Upon ligand binding, integrins cluster and recruit the adaptor proteins, talin, vinculin, and paxillin, forming focal adhesion complexes that bridge the ECM to the actin cytoskeleton [54]. This assembly triggers activation of FAK, which phosphorylates at Tyr397 and recruits Src family kinases thereby regulating cytoskeletal dynamics and promoting cell migration [55]. Parallel activation of the MAPK/ERK pathway regulates gene expression that modulates proliferation and differentiation, while the PI3K/Akt pathway promotes cell survival in the stressful microenvironment of injured tissues [56]. This mechanism is presented in Figure 2.

These interconnected pathways work synergistically to coordinate MSC responses during neuronal repair. For example, using a 3D culture model, Zeng et al., showed that Schwann cell-derived fibronectin accumulation within an MSC-containing gelatin sponge caused their neural differentiation, which resulted in improved nerve fibre generation in a rat spinal cord transection model. The development of thicker, authentic, and patent fibres was integrin-1α-dependent [57]. Further mechanistic detail was provided by Ma et al., who showed that sodium alginate/collagen + bone marrow-derived MSC hydrogels loaded with stromal cell-derived factor-1 (SDF-1) protected rats following experimental TBI, from motor and cognitive dysfunction whilst reducing inflammation and stimulating neurogenesis in a CXCR4-FAK/PI3K/AKT-dependent pathway [58]. Therefore, 3D dynamic control of these processes is critical for effective rebuilding of the neural ECM-dependent niche after injury.

### 4.2. Bidirectional Remodeling Promotes Survival in the Regenerative Niche

MSCs actively remodel their surrounding matrix by synthesizing new ECM components and producing MMPs that mediate controlled ECM remodeling by degrading damaged ECM components. This process facilitates their (MSC) migration as well as axonal extension, local stimulation of angiogenesis, and the deposition of newly synthesized ECM [54]. This cellular remodeling activity is responsive to the biochemical and mechanical cues provided by the scaffold. TGF-β, FGF-2, and platelet-derived growth factor (PDGF) growth factors sequestered within the matrix are released during remodeling, creating feedback loops that further modulate MSC behavior [59]. The scaffold provides instructive signals that determine MSC differentiation, whilst the ECM composition dictates which integrin receptors are engaged, and which downstream signaling cascades become activated [60]. For example, immortalised murine RAW 264.7 macrophages encapsulated within PEG-SH_4_ modified-HMW-HA secreted a strong anti-inflammatory phenotype in 3D cultures exposed to LPS, whilst low molecular weight HA oligomers had no effect [61].

For neural applications, scaffolds containing laminin and tenascin-C engage β1 integrin-containing receptors that promote neural stem cell proliferation and neuronal differentiation, as reviewed by [62]. Schaberg et al. showed that neural stem cell progenitor cells obtained from tenascin-C knockout mice had significantly delayed maturation and capacity to form neurospheres in culture compared to wild-type-extracted cells [63]. However, this effect was reduced when the cells were grown on a tenascin-rich substrate, and the stem cells showed increased migration track lengths, suggesting a critical modulation of motility that would likely impact regenerative capacity and overall spatially defined connectivity in the subependymal zone in an in vivo micro-environment.

In a mouse model of cerebral ischemic stroke, neuroblast chain formation and mi-gration along blood vessels were also shown to depend on β1 integrin signaling, which mediated adhesion to laminin and facilitated efficient soma translocation. Importantly, laminin-containing scaffolds enhanced neuroblast aggregation and directed migration toward the lesion site. These data indicate a potential role for ECM-mimetic substrates in promoting endogenous neuronal repair [64]. Similarly, Isik et al. reported the development of a neuronal differentiation guided HA scaffold functionalized with tyramine [65]. Using this tunable platform, they were able to guide induced human pluripotent stem cells to create neural organoids, with transcriptomic analysis and immunohistochemistry confirming functional lineage differentiation and fibrous morphologies, respectively.

This continuous cross-talk between MSCs (and indeed other stem cells) and their engineered microenvironment is able to sustain a self-reinforcing regenerative cycle. Within this cycle, the scaffolds first direct MSC differentiation toward neural lineages, MSCs then remodel the scaffold to create a more permissive microenvironment, and this remodeled matrix then further supports neurogenesis and axonal extension (see Table 2).

## 5. Implications for ‘Therapeutic’ Neural Regeneration

The ideal scaffold for dynamic protection and repair after brain injury should be instructive, and time-responsive, and be able to mimic a specific neuroregenerative niche. Based on the published knowledge to date, the hydrogel should be able to incorporate and sustain a high water-content, ideally including HA, collagen, GelMA, or chitosan–HA. This would most effectively mimic brain ECM and support MSC survival. It should have tunable stiffness and viscoelasticity to regulate mechanotransduction, and this would be transferred through positive modulation of integrin–FAK, Rho/ROCK, and YAP/TAZ pathways. In addition, it should possess adhesive motifs for example, RGD, laminin, collagen IV, or N-cadherin-mimetic sequences that will enhance MSC retention, survival, and neural-lineage signaling. These details have been described earlier in this review.

For real-time dynamic tuning, the scaffold should also include a capacity to enable controlled sequential release, which includes an early anti-inflammatory/protective phase followed by a later regenerative phase. In the acute phase, it should release or present factors microglial supressing factors particularly TSG-6, IL-10, TGF-β, TIMP3, miR-146a-enriched extracellular vesicles, and anti-NLRP3/anti-NF-κB signal blockers to suppress microglial pyroptosis, and secondary degeneration [70]. In the later phase, the same matrix should shift toward regeneration by delivering BDNF, NGF, GDNF, VEGF, IGF-1, MSC-derived exosomes, and/or secretome components that will promote neurogenesis, and support integration via synaptogenesis, axonal sprouting and remyelination. These molecules should also optimise the processes of angiogenesis, whilst dampening anti-inflammatory capacity in order to achieve the best neural circuit remodeling.

Recently, Zhang et al., confirmed this potential therapeutic value when they found that delivery of EVs from human umbilical cord-derived MSCs, primed with memantine stabilised the ECM and reduced stroke volume in a prothrombotic stroke mouse model [71]. The presence of memantine also improved neurological behavioural scores (e.g., better performance in the Morris water Maze), whilst protecting cells against apoptosis, when compared with unprimed MSC delivery. Similarly, neural stem cell-derived exosomes incorporated into an Exo- hydroxypropyl methylcellulose (HPMC) hydrogel significantly improved stroke recovery in C57BL/6 mice, subjected to MCAO, following stereotactic injection within the lesion. The gel maintained notably longer viability and secretion, when compared with cell injection alone [72].

**Figure 3:**
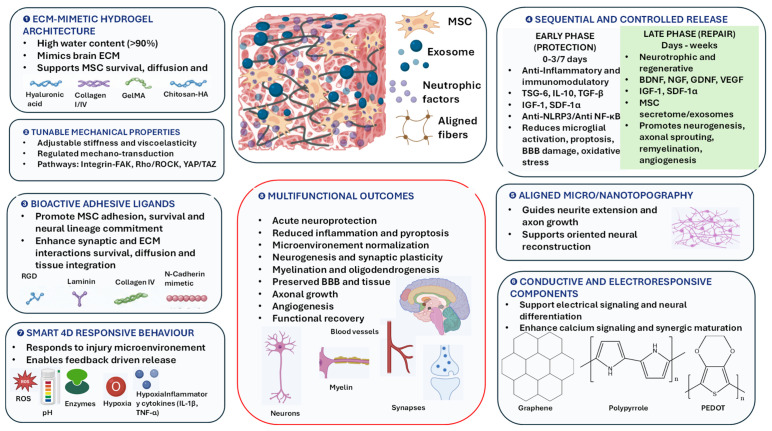
Engineered neuroregenerative hydrogels: integrating mechanotransduction, controlled release, and biomimetic cues for neural repair. Abbreviations: ECM—extracellular matrix, MSC—mesenchymal stem cell, GelMA—gelatin methacryloyl, HA—hyaluronic acid, FAK = focal adhesion kinase, YAP = Yes-associated protein, TAZ = transcriptional co-activator with PDZ-binding motif, RGD = Arginine–Glycine–Aspartic acid peptide motif, ROS = reactive oxygen species, IL-1β = interleukin-1 beta, TNF-α = tumor necrosis factor alpha, IGF-1 = insulin-like growth factor 1, SDF-1α = stromal cell-derived factor 1 alpha, BDNF = brain-derived neurotrophic factor, GDNF = glial cell line-derived neurotrophic factor, PEDOT = Poly(3,4-ethylenedioxythiophene). Created in BioRender. Tero-Vescan, A. (2026) https://BioRender.com/bmxf2v4.

Structurally, the scaffold should include aligned micro/nanotopography, such as electrospun fibers or patterned GelMA, to guide neurite extension and axonal orientation. A conductive component, such as polypyrrole, graphene, or other electroresponsive nanomaterials, would further support neural differentiation, calcium signaling, and bioelectrical integration. Finally, the most advanced version would be a smart time-responsive 4D scaffold, defined as a dynamic biomaterial capable of changing its structural, mechanical, or biochemical properties over time (the fourth dimension) in response to injury signals such as ROS, pH, enzymes, hypoxia, or inflammatory cytokines. This would enable real-time feedback-driven release of protective and regenerative cues [73]. In short, the ideal matrix would combine HA-rich ECM mimicry, MSC/exosome loading, adhesive ligand presentation, tunable mechanics, aligned architecture, controlled trophic release, conductive signaling, and inflammation-responsive behavior to move the injured brain from damage containment toward true repair. These multifunctional interactions are illustrated in Figure 3.

To date, there are only a few publications that have characterised time-release (4D) modulation of the ECM in neurological conditions. Zhang showed that GelMA hydrogel infused with adipose-derived MSCs, enabled sustained (30-day) production and secrotome secretion of EVs, which substantially reduced glial inflammation and concomitantly improved recovery in mice subjected to TBI [74]. We have already described the importance of servicing multiple or complex survival pathways during the acute and chronic phases of tissue damage, and to evaluate this, a cocktail of hydrogels composed of GelMA, which has both biodegradability and cell adhesion properties, together with a second gel composed of BDNF, GDNF, and cAMP (BGA) was tested for its ability to promote neuronal cell differentiation. In normal Wistar rats, stereotactic injection of BGA-GelMA containing human neural stem cells produced a more effective recovery in cortical experimentally induced TBI. Furthermore, the cocktail facilitated establishment of proper, functional neural-synaptic networks within the damaged lesion, together with increased interneuron regeneration [75]. Finally, Wu et al., created 4D mixed gradient hydrogels from photocrosslinkable alginate methacrylate (AlgMA) and GelMA, infused with microspheres containing a cocktail of growth factors, including TGF-β, IGF-1, PDGF-BB et al., which enabled secretion of the secretome and ECM-glycosaminoglycan production consistently over a prolonged time, of several weeks [76]. In addition, they showed the capacity for the gel to modify/change its shape in the presence of co-infused fibroblasts, indicating potential biomimetic use for tissue and organ replacement. Whist still at the early ‘research’ stages, this technology may have future interest for neurological therapy and repair.

## 6. Translational Capacity: A Brief Mention of Future Perspectives and Current Limitations

Following CNS injury, the tissue microenvironment is initially dominated by overexpression of pro-inflammatory signals, enhanced reactive gliosis, and a blockage of inhibitory ECM molecules, particularly chondroitin sulfate proteoglycans (CSPGs) [60]. Shifting the balance via dynamic reciprocity would provide a greater abundence of pro-regenerative adhesion substrates that would compete with inhibitory signals and present neurotrophic factors at appropriate concentrations. The resulting optimised mechanical cues would preferentially maintain neuronal differentiation over astrocytic scarring, and supporting MSC survival and paracrine function to sustain immunomodulatory effects.

**Figure 4:**
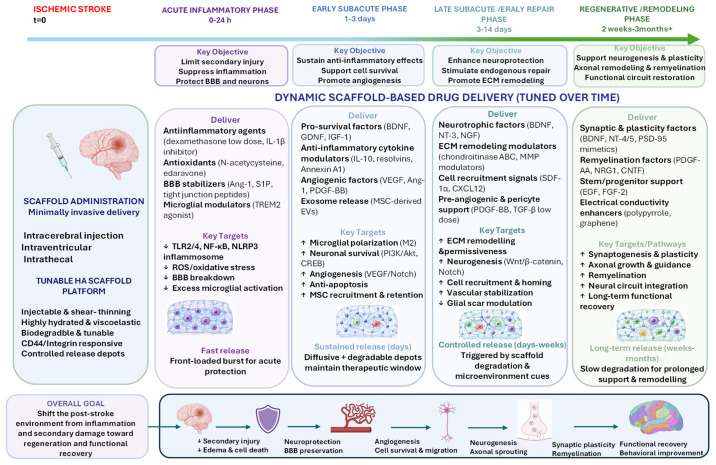
Algorithmic scheme of the stage-dependent response of an injectable, HA-rich hydrogel loaded with MSCs/exosomes in ischemic stroke Abbreviations: MSC = Mesenchymal Stem Cell, HA—hyaluronic acid, BBB—blood–brain barrier, NF-κB—nuclear factor kappa-light-chain-enhancer of activated B cells, NLRP3—NOD-like receptor family pyrin domain containing 3, TSG-6—tumor necrosis factor-stimulated gene-6, IL-10—interleukin-10, TIMP3—tissue inhibitor of metalloproteinases 3, miR-146a—microRNA-146a, BDNF—brain-derived neurotrophic factor, GDNF—glial cell line-derived neurotrophic factor, IGF-1—insulin-like growth factor 1, VEGF—vascular endothelial growth factor, Ang-1—angiopoietin-1, PDGF-BB—platelet-derived growth factor-BB, CD44—cluster of differentiation 44, YAP—Yes-associated protein, TAZ—transcriptional co-activator with PDZ-binding motif, ECM—extracellular matrix. Created in BioRender. Tero-Vescan, A. (2026) https://BioRender.com/c0qi1u9.

Native neurogenic niches, such as the subventricular zone, contain critically important specialized ECM structures (fractones) composed of laminin, collagen IV, and heparan sulfate proteoglycans that regulate neural stem cell proliferation and differentiation. Therefore, ECM-mimetic scaffolds should be designed to recapitulate these features, providing MSCs and endogenous neural progenitors with the signals necessary for neurogenesis and circuit reconstruction [77]. The example in Figure 4, shows how such a scaffold could be generated that could optimise tissue protection and recovery after acute ischaemic stroke.

The evidence provided in this review supports the concept of a mutual support mechanism in MSC-scaffold interactions with roles in signaling, adhesion, mechanotransduction, and topographic guidance. At the same time the biomaterial scaffolds actively direct MSC behavior towards neuroprotection, neurogenesis, and circuit reconstruction. Therefore, scaffold-cell interactions should be considered as a multidimensional synergistic mechanism essential in the transition of the injured CNS from a pro-inflammatory state to a regenerative niche. The most important medical application of this interaction would be the design of next-generation biomaterials that more effectively support functional recovery after neuronal injury.

Although dynamically tuned MSC-based scaffolds are very promising form a therapeutic perspective, the clinical translation is limited by substantial regulatory and manufacturing challenges. Complex biomaterials incorporating cells, extracellular vesicles, or bioactive molecules require extensive preclinical validation, standardized GMP manufacturing, and approval by regulatory agencies such as the FDA or EMA before clinical use. Administration of non-approved biomaterials outside authorized clinical trials is not permitted, making regulatory compliance one of the major barriers to clinical translation.

From a translational perspective, hydrogel-based MSC scaffolds currently represent the most clinically advanced biomaterial platform for CNS repair, because of their excellent biocompatibility, injectability, and ability to mimic the native ECM. However, the strongest evidence supporting their efficacy remains preclinical, from rodent models of stroke, traumatic brain injury, and spinal cord injury (see Table 3). Early-phase clinical studies have established the safety of several hydrogel formulations, whereas robust evidence demonstrating consistent functional benefit in patients is still lacking. Therefore, although hydrogel scaffolds constitute one of the most promising translational strategies in neural tissue engineering, multifunctional dynamically tuned scaffolds incorporating living cells, extracellular vesicles, or programmable release systems should currently be regarded as emerging technologies rather than established clinical therapies.

**Table 3:** Current Level of Evidence of Hydrogel-Based MSC Scaffolds.

Clinical Application	Evidence for Hydrogel-Based MSC Scaffolds	Clinical Status	Representative References
Skin wound healing	Strong preclinical evidence; several early clinical studies; hydrogel dressings widely used clinically, MSC-hydrogel constructs remain investigational	Early clinical evaluation	[78]
Bone repair	Extensive preclinical evidence with encouraging early clinical studies in critical-sized bone defects	Early clinical evaluation	[79]
Myocardial infarction	Robust preclinical evidence; several Phase I/II clinical trials evaluating MSC-containing biomaterial platforms	Phase I/II clinical trials	[80]
Spinal cord injury	Strong efficacy in rodent and large-animal models; limited first-in-human studies	Early clinical trials/predominantly preclinical	[81]

## 7. Future Perspectives

Although promising effects have been shown in vivo for MSC-based multifunctional scaffolds and HA-enriched biomaterials in improving CNS response to injury, several important limitations exist and these must be addressed before they can even be considered for use in human medicine. Almost all of the current evidence comes from small (rodent) animal studies, and these have been elucidated using many different scaffold compositions, cell sources, dosing strategies, routes of administration, injury models, and outcome measurements. This limits direct comparison and translational reproducibility.

Many experimental studies report improvements in neuroinflammation, angiogenesis, axonal remodeling, and behavioral recovery. However, the precise mechanisms of long-term scaffold integration, their biodegradation kinetics, immune compatibility, and phenotype stability in the injured human brain are not completely understood.

An important aspect is that there have been very few clinical trials and essentially no large-scale randomized clinical trials evaluating dynamically tunable MSC scaffold systems, particularly in neurological diseases, stroke, traumatic brain injury, or spinal cord injury. Consequently, the long-term safety, feasibility, standardization of manufacturing techniques, and therapeutic efficacy of these scaffolds in human neurological disorders remain debatable. Therefore, further translational studies and carefully designed clinical trials will be essential before such regenerative scaffold systems can be considered viable therapeutic strategies for routine neurological practice.

## Figures and Tables

**Table 2:** Key regulatory axes governing scaffold–MSC interactions and their impact on cellular responses in neural tissue engineering.

Regulatory Axis	Scaffold Contribution	MSC Response	Ref
Adhesion-dependent signaling	Ligand presentation (RGD, laminin epitopes)	Integrin clustering, FAK activation, cytoskeletal reorganization	[66]
Mechanotransduction	Defined stiffness, viscoelasticity, and dynamic mechanical cues	Integrin FAK activation, cytoskeleton tension (RhoA/ROCK), YAP/TAZ nuclear translocation, lineage commitment	[67]
Topographical guidance	Aligned nanofibers, micro/nanotopography mimicking ECM	Contact guidance, β1-integrin focal adhesion formation, directed neurite extension, enhanced synaptogenesis	[68]
Biochemical stimulation	Growth factor incorporation, controlled release of cytokines and trophic factors (e.g., BDNF, NGF, VEGF, IGF-1)	Paracrine signaling, neurotrophin secretion, immunomodulation, enhanced cell survival and tissue repair	[69]

Abbreviations: RGD—Arg–Gly–Asp, FAK—focal adhesion kinase, RhoA—Ras homologous GTPase, ROCK—Rho-associated coiled-coil containing protein kinase, YAP—Yes-associated protein, TAZ—transcriptional co-activator with PDZ-binding motif, ECM—extracellular matrix, BDNF—brain-derived neurotrophic factor, NGF—nerve growth factor, VEGF—vascular endothelial growth factor, IGF-1—insulin-like growth factor 1.

## Data Availability

No new data were created or analyzed in this study. Data sharing is not applicable to this article.

## Abbreviations

The following abbreviations are used in this manuscript:

6-OHDA	6-hydroxydopamine
ADSCs	adipose-derived stem cells
Akt	protein kinase B
BBB	blood–brain barrier
BDNF	brain-derived neurotrophic factor
BMP-2	bone morphogenetic protein 2
CD44	cluster of differentiation 44
CNS	central nervous system
COL2	collagen type II
CSPGs	chondroitin sulfate proteoglycans
DALYs	disability-adjusted life years
DAMP	damage-associated molecular pattern
ECM	extracellular matrix
ERK1/2	extracellular signal-regulated kinase 1/2
FAK	focal adhesion kinase
FGF-2	fibroblast growth factor 2
GDNF	glial cell line-derived neurotrophic factor
GelMA	gelatin methacryloyl
GNPs	graphene nanoplatelets
GSDMD	gasdermin D
HA	hyaluronic acid
hBMSCs	human bone marrow-derived stem cells
hDPSCs	human dental pulp-derived stem cells
HIF-1α	hypoxia-Inducible Factor 1 alpha
HMW	high molecular weight
hPDLSCs	human periodontal ligament-derived stem cells
hUMSCs	human umbilical cord-derived MSCs
ICAM-1	intercellular adhesion molecule 1
IGF-1	insulin-like growth factor 1
iNOS	inducible nitric oxide synthase
IRAK1	interleukin-1 receptor-associated kinase 1
LMW	low molecular weight
LPS	lipopolysaccharide
LYVE-1	lymphatic vessel endothelial hyaluronan Receptor 1
MAP2	microtubule-associated protein 2
MAPKs	mitogen-activated protein kinases
MFAT	microfragmented adipose tissue
MMP	matrix metalloproteinase
MSCs	mesenchymal stem cells
MyD88	myeloid differentiation primary response 88
NF-κB	nuclear factor kappa-light-chain-enhancer of activated B cells
NGF	nerve growth factor
NLRP3	NOD-like receptor family pyrin domain containing 3
p38MAPK	p38 mitogen-activated protein kinase
Pax6	paired box gene 6
PDGF	platelet-derived growth factor
PI3K	phosphoinositide 3-kinase
PPy/PLA	polypyrrole/polylactide
PSD-95	postsynaptic density protein 95
RGD	Arg–Gly–Asp
RHAMM	receptor for hyaluronan-mediated motility
Rho	Ras homologous GTPase
RhoA	Ras homolog family member A
ROCK	Rho-associated coiled-coil containing protein kinase
RUNX-2	runt-related transcription factor 2
SDF-1	stromal cell derived factor-1
Sox2	SRY-box transcription factor 2
Sox9	SRY-box transcription factor 9
Src	proto-oncogene tyrosine-protein kinase
STAT1/3	signal transducer and activator of transcription 1/3
TAZ	transcriptional co-activator with PDZ-binding motif
TGF-β	transforming growth factor beta
TIMP3	tissue inhibitor of metalloproteinase-3
TLR2/4	toll-like receptor 2/4
TNF-α	tumor necrosis factor alpha
TRAF6	TNF receptor-associated factor 6
TSG-6	tumor necrosis factor-stimulated gene-6
Tuj1	βIII-tubulin
UHMW	ultra-high molecular weight
VEGF	vascular endothelial growth factor
VEGFR2	vascular endothelial growth factor receptor 2
WJ-MSCs	Wharton’s jelly-derived mesenchymal stem cells
YAP	Yes-associated protein
